# Neonatal Duodenal Duplication Cyst

**Published:** 2014-01-01

**Authors:** Haidar AM, Elgharmool BM

**Affiliations:** Pediatric and Neonatal Surgical Department, Tripoli's Medical Center, Tripoli, Libya

**Dear Sir**

A 2800 gram baby boy was born with a prenatal diagnosis of a choledochal cyst; he was admitted to NICU for further evaluation. Clinically he was jaundiced with a palpable abdominal mass in the right hypochondrium. Choledochal and duplication cysts were differential diagnoses. Ultrasonography by an experienced sonographer or a CT scan is usually sufficient for diagnosis. Communicating duplication cyst can easily be diagnosed on barium meal follow through which may show contrast in the cyst; isotope scanning may show areas with ectopic mucosa if lining the cyst.[1-3] In this case, the CT scan with intravenous contrast showed an intraperitoneal cystic lesion (10cm x 7cm x 6cm) in diameter extending from the sub hepatic region down to the pelvic inlet, with a thin non-enhancing wall and fluid attenuation with a significant mass effect on adjacent structures and bowel.

At explorative laparotomy a duodenal duplication cyst at the second part was found (Fig.1) and a complete resection and anastomosis was not an option. The cyst content was non-bilious fluid. The cystic wall was opened and excised up to the common wall with stripping of lining mucosa. The baby recovered uneventfully and discharged home at the age of 19 days. Histopathology was consistent with duodenal duplication cyst with an ectopic gastric mucosa. On follow up the infant is doing well and thriving.

**Figure:**
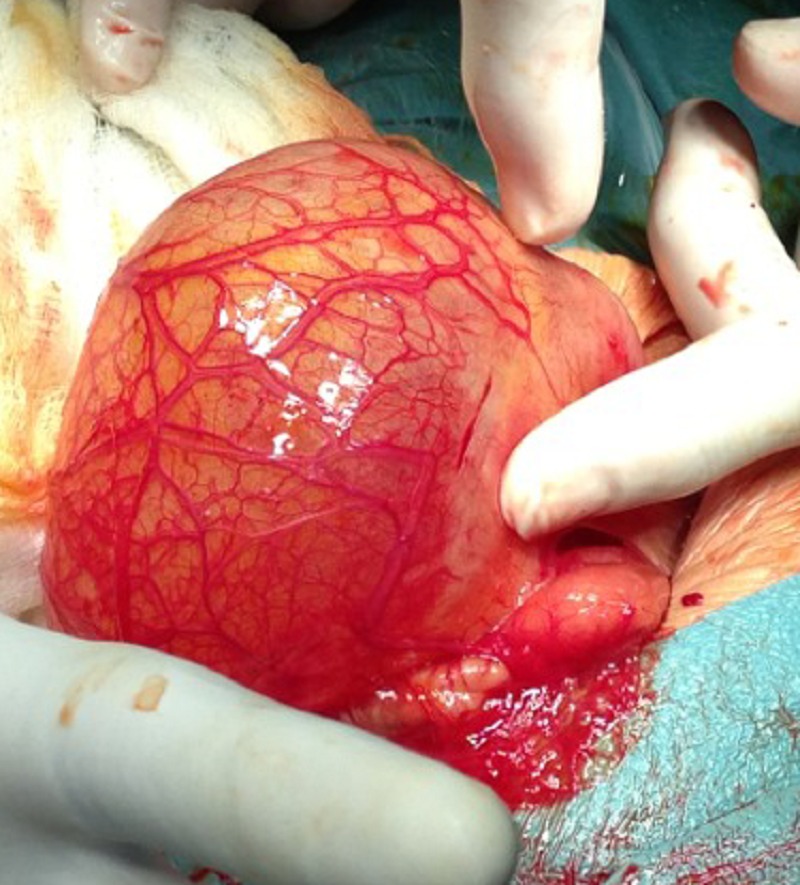
Figure 1:Duplication cyst

Treatment of alimentary tract duplication cysts is total surgical excision but it should be tailored according to the site. In difficult sites like in our case, subtotal excision with mucosal stripping should be done.[1, 2]

## Footnotes

**Source of Support:** Nil

**Conflict of Interest:** None

